# Chikungunya in 2025: Comprehensive Insights into Virology, Diagnostics, Vaccines, and Antiviral Therapies

**DOI:** 10.3390/v18010100

**Published:** 2026-01-12

**Authors:** Zeyong Zheng, Hua Ji, Zeping Shan, Jiangcheng Xu, Jiarui Li, Xueting Zhang, Jiajia Zheng, Shibo Jiang, Naru Zhang

**Affiliations:** 1 School of Medicine, Hangzhou City University, Hangzhou 310015, China; 32204251@stu.hzcu.edu.cn (Z.Z.); 32204023@stu.hzcu.edu.cn (H.J.); 32204159@stu.hzcu.edu.cn (Z.S.); 32504227@stu.hzcu.edu.cn (J.X.); 32304005@stu.hzcu.edu.cn (J.L.); 32304185@stu.hzcu.edu.cn (X.Z.); 2 Department of Respiratory and Critical Care Medicine, People’s Hospital of Anji, 699 Tianmu Road, Anji County, Huzhou 313300, China; 3 Shanghai Institute of Infectious Disease and Biosecurity, School of Basic Medical Sciences, Fudan University, Shanghai 200032, China

**Keywords:** CHIKV, invasion mechanism, diagnostic techniques, vaccines, drugs

## Abstract

Chikungunya virus (CHIKV) is a mosquito-borne alphavirus prevalent in more than 110 countries and regions, including Africa, Asia, the Americas, and Europe. It can cause acute fever, rash, and severe joint pain, and some patients may develop chronic arthritis, which significantly impairs quality of life. CHIKV infection can occasionally be fatal, with neurologic disease a particularly severe manifestation. Following its resurgence in 2005, CHIKV has emerged as a major threat to global public health. This review summarizes diagnostic techniques, advances in vaccine development, and the latest drug interventions for CHIKV. We also present an overview of the epidemiology, structure, and invasion mechanisms of epidemic hotspots in 2024–2025 and propose evidence-based strategies for effective prevention and control of CHIKV infection.

## 1. Introduction

Chikungunya virus (CHIKV) is a mosquito-borne virus belonging to the Aphavirus genus within the *Togaviridae* family. It was first isolated and identified in Tanzania in 1952. Over the last two decades, it has once again become a key pathogen threatening global public health [1,2]. The virus is primarily transmitted by *Aedes aegypti* and *Aedes albopictus* mosquitoes. Infection can cause acute febrile illness, with typical clinical features including high fever, severe polyarthritis, and rash [3]. Notably, up to 60 percent of patients develop persistent, disabling joint pain that lasts for months to years. Although CHIKV infection has a low fatality rate, periodic epidemics impose a substantial socioeconomic burden driven by workforce loss, increased pressure on healthcare systems, and chronic sequelae, all against the backdrop of an aging population and the rising prevalence of comorbidities [4].

While CHIKV infection is underdiagnosed in many parts of the world, it underwent an unprecedented resurgence from 2024 to 2025 [4]. The World Health Organization (WHO) received reports of over 1.6 million suspected cases, including China’s largest local outbreak, with more than 160,000 laboratory-confirmed cases. By September 2025, a total of 2197 suspected cases and 108 laboratory-confirmed cases of CHIKV infection were reported across Africa. By 3 October 2025, a total of 56,456 CHIKV cases, including 40 deaths, were also reported in four European countries and regions, including France, Italy, Reunion, and Mayotte. CHIKV prevalence in Asia is primarily concentrated in Southeast Asia, Central Asia, South Asia, and the Western Pacific subregion of East Asia [5]. Over the past two years, CHIKV case numbers have increased in Pakistan, Indonesia, and Bangladesh. By 27 September 2025, Guangdong province in China reported more than 16,000 laboratory-confirmed cases of local CHIKV transmission, marking the largest local Chikungunya fever outbreak in China’s history. By 15 November, 48 new local cases were reported within the province. CHIKV cases in Guangdong province have now been reported in 21 cities, mainly concentrated in Foshan (10,040), Jiangmen (5223), Guangzhou (590), Shenzhen (140), Zhanjiang (112), Zhuhai (60), and Zhongshan (54). Among all reported cases, young adults aged 18–45 years accounted for the highest proportion [6].

## 2. Virology and Pathogenic Mechanisms of CHIKV

### 2.1. Genome, Protein Structure, and Function of CHIKV

**Figure 1:**
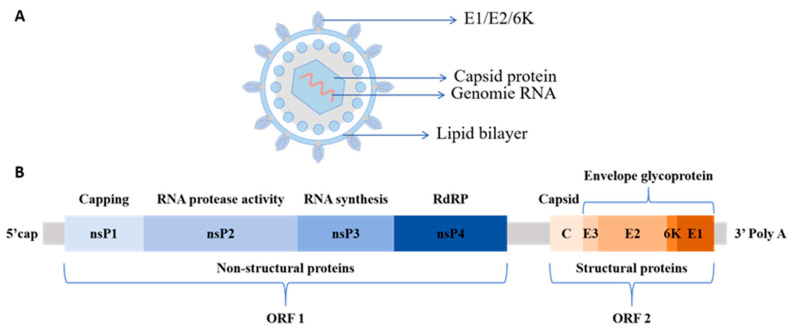
Schematic diagram of CHIKV and its genome. (**A**) Structure of the CHIKV particle, an enveloped spherical structure with a lipid bilayer on the outer layer and enveloped glycoproteins, such as E1, E2, and 6K, embedded in the membrane. Genomic RNA, both single-stranded and positive-sense, is wrapped by icosahedral capsid proteins on the inside. (**B**) The genomic structure of CHIKV contains two ORFs. ORF1 encodes nsP1-nsP4, which are responsible for replication-related functions, such as RNA capping, synthesis, and protease activity. ORF2 encodes five structural proteins, including C, E3, E2, 6K, and E1, which are involved in the assembly and envelope formation of viral particles.

CHIKV particles are spherical, with a diameter of approximately 60–70 nm. They have a lipid envelope and a nucleocapsid core composed of single-stranded positive-sense RNA and capsid proteins with only one serotype [7]. The viral genome is approximately 11.8 kb in length, and the coding region contains two open reading frames (ORFs), ORF1 and ORF2. ORF1, which is located at the 5′ end of the genome, constitutes two-thirds of the total length and encodes four non-structural proteins (nsPs), nsP1 to nsP4, in sequence. ORF2 at the 3′ end encodes five structural proteins, including nucleocapsid protein (C), envelope glycoproteins E1, E2, E3, and 6K protein (Figure 1) [8,9].

**Figure 2:**
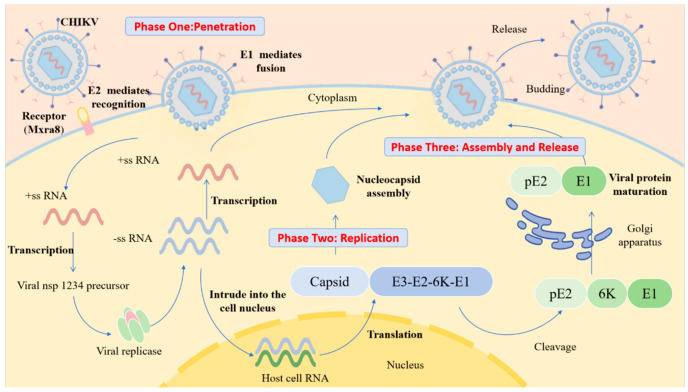
The process of CHIKV infection. E2 protein mediates specific recognition and binding of virus to host receptor (Mxra8), driving the virus to attach to the cell surface. E1 protein mediates fusion of the viral envelope with the host cell membrane, releasing the viral +ssRNA genome into the cytoplasm. Viral +ssRNA is directly translated into nsP precursors, assembled into replication complexes, transcribed to generate -ssRNA, and used as a template to synthesize progeny +ssRNA. At the same time, +ssRNA is translated to produce structural protein precursors. After the structural protein precursor is cut and processed, the capsid protein binds to the progeny genomic RNA (gRNA) to form the nucleocapsid, which then interact with the E2/E1 glycoprotein complex localized on the host cell membrane to acquire an envelope, followed by the extracellular release of the mature virus.

CHIKV nsPs are key molecules that mediate virus–host cell interactions and regulate pathogenic mechanisms. Each protein exhibits highly specific functions [10]. The nsP1 performs both N7-guanine methyltransferase (MTase) and guanylate transferase (GTase) activities, which catalyze the formation of the 5′ cap structure of nascent viral RNA and are critical for maintaining viral RNA’s stability [11]. Additionally, the palmitoylation of nsP1 enables it to target cholesterol-rich microdomains on the host cell membrane, providing key sites for the assembly of viral replication complexes [12]. The nsP2 is the largest nsP encoded by the alphavirus genome. It features an RNA-specific helicase domain at its N-terminus and a cysteine protease domain at its C-terminus. This protease cleaves the viral polyprotein precursor into functional replicase components (nsP1, nsP2, nsP3, nsP4), a key step in the viral replication cycle [13]. The nsP3 protein has a modular domain structure. The N-terminus, which exhibits ADP-ribose hydrolase (MAR hydrolase) and phosphatase activities, participates in viral replication by regulating nucleic acid metabolism [14]. The C-terminal contains a hypervariable domain (HVD) that binds to multiple signaling molecules in the host cell, mediates virus–host interactions, and regulates host cell physiological functions to support viral replication [15]. It also contains the alphavirus-unique domain (AUD), which plays an indispensable role in viral genome replication and transcription. The nsP4 is the core catalytic subunit for viral RNA replication. Its RNA-dependent RNA polymerase (RdRp) domain efficiently catalyzes viral RNA synthesis. It is a key enzyme for viral genome replication and subgenomic RNA transcription, playing a central regulatory role in viral replication [16,17]. CHIKV structural proteins play distinct roles in viral assembly and infection.  As shown in Figure 2, the C protein, a core component of assembly, contains an N-terminal RNA-binding domain that facilitates specific binding between the viral genome and C protein, ensuring RNA capping and nucleocapsid formation [18]. The C-terminal contains a protease domain, which promotes viral release through interactions with the E2 protein. The E1 protein mediates fusion between the viral envelope and the host cell membrane. The E2 protein mediates specific interactions between the virus and host cell receptors, participates in viral attachment, recognition, and binding, and facilitates the entry of viral RNA into the host cell via endocytosis. The E3 protein promotes the correct folding of the E2 protein precursor and stabilizes E1/E2 trimer conformation. The 6K protein as well as its translational frameshift product, transferase (TF), form ion channels and participates in viral release by promoting vesicle fusion within the endoplasmic reticulum [19,20].

### 2.2. Infection Mechanism of CHIKV

CHIKV’s cell invasion pathways are cell-type-specific, and the associated host cell receptors and binding molecules exhibit substantial diversity [21,22]. Among these, matrix remodeling-associated protein 8 (Mxra8) is one of the most well-characterized human CHIKV receptors. It is widely expressed on the surface of epithelial and mesenchymal cells [23,24]. Both in vitro cell-based assays and animal model studies have confirmed that Mxra8 expression levels are positively correlated with CHIKV infection efficiency. Furthermore, joint swelling and the degree of CHIKV infection are significantly reduced in Mxra 8-deficient mice [25]. Notably, the absence or low expression of Mxra8 does not completely block CHIKV infection, indicating the presence of alternative receptors in the host. CD147, also known as basigin or EMMPRIN, is another validated CHIKV receptor widely expressed in fibroblasts, endothelial cells, and other cell types. This molecule shares high structural homology with Mxra8, but its specific molecular interactions with CHIKV proteins remain to be further clarified [26]. Mediated by E2 protein, CHIKV recognizes and binds to the host cell surface. It then enters target cells via clathrin-mediated endocytosis, where it interacts with Ras-related protein Rab-5A(RAB5)-positive endosomes and undergoes E1 protein-mediated membrane fusion [27]. The fusion process is triggered by endosomal acidification, and the presence of cholesterol in the target membrane significantly augments fusion efficiency. It has been confirmed that the cholesterol-depleting agent methyl-β-cyclodextrin and lysosomotropic drugs, e.g., chloroquine and baflomycin, that inhibit endosomal acidification can effectively suppress CHIKV infection [28].

Micropinocytosis is also an important alternative pathway for CHIKV infection of human muscle cells [29]. This process is regulated by a complex signaling cascade. Following viral binding to the cell membrane, it activates signaling molecules, such as receptor tyrosine kinases (RTKs). These RTKs then trigger intracellular signaling cascades that induce actin cytoskeleton rearrangement, leading to the formation of irregular folds and vesicular structures. Upon collapse of these structures, they engulf the virus–receptor complex and liquid-phase macromolecules to form macropinosomes, completing viral internalization. This process relies on signal transduction from molecules, including phosphatidylinositol 3-kinase (PI3K), protein kinase C, the Rho GTPase Rac1, and actin polymerization-mediated macropinosome formation. The synergistic effects of multiple entry pathways enable CHIKV to invade various cell types across different tissues, allowing for extensive dissemination within the host [30].

### 2.3. Mechanism Underlying CHIKV Immune Escape

After CHIKV infection, its viral RNA can be specifically recognized by host pattern recognition receptors, such as toll-like receptor (TLR) 3, TLR7, and TLR8, resulting in the activation of interferon regulatory factors (IRFs) and NF-κB signaling pathways. These pathways induce type I interferon (IFN) and interferon-stimulating factor (ISG) expression and initiate host antiviral immune response [31]. During the acute phase of CHIKV infection, viral clearance mainly relies on innate immunity’s early response (including the IFN family and innate immune cells) and clearance through adaptive immunity, supplemented by the regulatory and synergistic effects of cytokines (e.g., pro-inflammatory factors IL-6 and TNF-α) which promote immune cell recruitment and activation. However, CHIKV has evolved targeted immune escape strategies that regulate host immune signals through nsPs to achieve efficient immune escape, which depends on the functional mediation of nsPs [32]. Specifically, nsP1 interacts directly with host cell cyclic GMP-AMP synthase (cGAS). Inhibiting the activation of the cGAS-stimulator of interferon genes (STING) signaling pathway blocks type I IFN production at the source, significantly weakening the host’s innate immune defense. The nsP2 blocks the host’s antiviral response through a dual mechanism. On the one hand, it guides RNA polymerase II in the nucleus to catalyze the protease degradation of subunit Rpb1, inhibiting host cell gene transcription and reducing antiviral gene expression. On the other hand, it blocks the downstream transmission of the IFN signaling pathway by promoting the re-output of signal transducer and activator of transcription 1 (STAT1) from the nucleus to the cytoplasm [33]. This prevents complete activation of the host’s innate and adaptive immune responses. With its ADP-ribohydroxy enzyme activity, nsP3 removes the ADP-nucleosylation modification of nsP2 and maintains a highly active state of nsP2 to assist the virus in evading host ADP-ribotransferase-mediated antiviral activity [19,34].

In addition to innate immune escape, CHIKV can also partially evade adaptive immune responses. Studies have shown that the virus can establish persistent infection in joint tissues, with the core mechanism linked to CD8^+^ T cell exhaustion. Cytotoxic molecule expression levels, such as granzyme B and perforin, in CD8^+^ T cells are significantly downregulated following infection, resulting in an impaired ability to kill infected cells and inefficient viral clearance, thereby enabling viral persistence [35].

## 3. Diagnosis of CHIKV Infection

The clinical manifestations of CHIKV infection are highly heterogeneous and nonspecific. Core symptoms, such as fever and arthralgia, often overlap with those of dengue virus (DENV), Zika virus (ZIKV), and other viral infections. Therefore, accurate diagnosis based solely on clinical manifestations is challenging, making laboratory testing crucial. Currently, laboratory testing for CHIKV includes nucleic acid testing (NAT), serological assays, and next-generation sequencing (NGS). NAT, represented by RT-PCR, can directly target viral nucleic acid for rapid diagnosis [36,37,38,39,40,41,42]. Serological assays are conducted based on IgM and IgG antibodies for the middle to late stages of infection, respectively, as well as epidemiological surveys [36,37,38,39,41,42,43,44,45].

Studies have demonstrated that RT-PCR is the gold standard for early detection of CHIKV infection, with a positive detection rate exceeding 90% within the first four days of illness [37]. A clinical study involving 646 cases demonstrated that RT-PCR detected 31 CHIKV-positive samples (4.79% positive rate), while simultaneous IgM antibody testing yielded only 1 positive result [45]. A retrospective study by Boonanek et al. further corroborated this outcome. They performed simultaneous RT-PCR and IgM antibody testing on 31 hospitalized children with suspected CHIKV infection. As a result, 30 cases were diagnosed via plasma RT-PCR, but only 1 case was positive for IgM antibodies [44]. Based on these results, RT-PCR remains the preferred testing method during the acute phase of CHIKV infection. Although nucleic acid amplification tests (NAATs) are the gold standard for diagnosing viral infections, serological assays have emerged as important supplementary diagnostic tools given the limitations of RT-PCR, such as a narrow viremic window, high cost, and dependence on specialized equipment [38]. Studies have demonstrated that RT-PCR exhibits the highest sensitivity during the early phase of CHIKV infection (days 1–4 of the disease’s course), with a positive rate of over 90%, whereas the IgM detection rate is only 3.2–13.3% during the same period. However, IgM’s positive rate increases significantly to 51.6% starting from day 5 of illness, and its sensitivity exceeds that of RT-PCR by day 6, reaching over 90% by day 10 [37]. The IgG antibody immune response is relatively delayed, and it is rarely detectable within days 1–9 of illness. The positive rates on days 10 and 12 are 50.0% and 82.0%, respectively, indicating that IgG antibodies are more suitable for identifying past infections [37]. Therefore, CHIKV diagnosis should adopt a phase-specific testing strategy whereby RT-PCR detection is preferred on days 1–4 but IgM detection is preferred starting from day 5, when the body starts producing detectable antibodies. This pattern could improve diagnostic accuracy throughout the entire course, reduce misdiagnosis, and accelerate accurate diagnosis [37].

In recent years, NGS, particularly metagenomic NGS (mNGS), has gradually emerged as an important tool in infectious disease diagnosis. This technology does not require a pre-specified target pathogen and can simultaneously detect a wide range of microorganisms, including viruses, bacteria, fungi, and parasites. Thus, mNGS can significantly improve diagnostic efficiency and accuracy, and it has been widely used in clinical research and practice [36,39,46]. NGS technology was used for CHIKV sequencing and analysis in studies of chikungunya fever outbreaks in India (2019–2022), Thailand (severe pediatric cases in Bangkok in 2019 and the epidemic during 2020–2023), and Bangladesh (2024). Circulating strains in each region belong to the East/Central/South African (ECSA) genotype (further denoted as the ECSA-IOL sublineage in Thai studies), and these circulating strains generally harbor the characteristic E1-K211E mutation. Thai studies also found that this mutation often coexists with the E2-V264A mutation. This technology is also used for CHIKV genetic variation analysis, as well as evolutionary tracing, e.g., distinguishing sublineages between Bangladeshi strains and the local 2017 strain. It has also been used for regional transmission pathway analysis, e.g., analyzing genetic similarity between Thai strains and those from neighboring countries.

NGS technology also provides molecular diagnostic support for etiological confirmation and epidemiological surveillance of clinical cases, including pediatric cases [37,39,40,44]. Another case study used NGS to sequence and analyze the viral genome in patients’ plasma and cerebrospinal fluid samples. The researchers identified a specific marker for the deletion of 62 amino acids in the nsP3 region through sequence alignment analysis, confirming the attenuated VLA1553 vaccine strain [36]. Furthermore, in January 2024, one imported CHIKV case was confirmed in Guangzhou, China. The patient’s serum exhibited an extremely low CHIKV viral load (Ct = 32.62), rendering it challenging to obtain the complete viral sequence via conventional culture and sequencing methods [47]. Employing an optimized mNGS approach, researchers successfully acquired the full-length viral genome (strain GZ01/2024). Phylogenetic analysis revealed that this strain belongs to the Asian lineage and shares high homology with circulating strains in Indonesia and Southeast Asia [47]. Combined with the patient’s travel history, the infection was definitively traced to Timor-Leste, highlighting the superiority of NGS technology in tracing imported cases with low viral loads [47].

Notably, CHIKV surveillance is inadequate in many tropical and subtropical countries and regions. Lack of diagnostic testing is a primary contributing factor. Significant discrepancies in the cost and infrastructure requirements of different detection methods have further widened the diagnostic gap across regions. NAT, typified through RT-PCR, offers high sensitivity but is associated with relatively high costs and stringent demands for laboratory facilities, specialized equipment, and skilled technicians. These conditions remain unattainable in many resource-limited areas. In contrast, serological testing features lower costs and more flexible equipment requirements. However, NGS technology is the most expensive detection modality, necessitating not only costly sequencing instruments but also specialized bioinformatics analysis platforms and technical teams, making it unsuitable for large-scale epidemic screening [46]. Collectively, these factors have led to global disparities in CHIKV surveillance and control, underscoring the urgent need for improvement through sustained technology dissemination, resource investment, and international collaboration.

## 4. Vaccine Development and Clinical Trial Dynamics for CHIKV

Research on CHIKV vaccines remains exploratory. Currently, two chikungunya vaccines approved by the U.S. Food and Drug Administration (FDA) are available in the U.S.A. One is the live attenuated vaccine IXCHIQ, but its use is restricted; the other is the virus-like particle (VLP) vaccine VIMKUNYA. Meanwhile, several potential vaccine candidates have progressed to preclinical development, and some have advanced further to clinical trials (Table 1). Currently, CHIKV vaccines under development can be classified by their technical platforms, such as inactivated vaccines, live attenuated vaccines, subunit vaccines, VLP vaccines, or recombinant viral vector-based vaccines [48].

### 4.1. FDA-Approved CHIKV Vaccines

The live attenuated vaccine IXCHIQ (VLA1553) is based on the ECSA genotype CHIKV strain LR2006- OPY1, which was isolated from Reunion Island in 2006. It achieves attenuated virulence by genetically modifying the virus to delete 62 amino acids at the C-terminus of the nsP3 and inserting the AYRAAAG linker sequence. The vaccine is produced by propagating the virus in Vero cells, followed by purification and lyophilization. However, in August 2025, the FDA urgently suspended its use in the U.S.A based on reports of multiple serious adverse events. It is currently available only in the European Union and Canada [72]. In addition, the vaccine is authorized for a limited population, excluding pregnant women and those with immune deficiencies, and requires careful assessment in post-vaccination adverse events during outbreak settings [36]. The VLP vaccine VIMKUNYA (PXVX0317) was developed based on the West African genotype CHIKV strain 37997 isolated from Senegal. During production, DNA plasmids encoding CHIKV structural proteins (capsid, E3, E2, 6K, and E1) are transfected into human embryonic kidney HEK293 cells to generate VLPs. These proteins autonomously assemble into genome-free VLPs, which are then purified via centrifugation and chromatography. Aluminum hydroxide adjuvant is added to some formulations to boost immunogenicity [61]. It is currently approved for use in people aged 12 and above, while safety data for children and pregnant women are still being accumulated. Furthermore, reactivity should be monitored in previously infected individuals. The incidence of swelling at the injection site after vaccination (10%) is significantly higher in baseline seropositive individuals than in seronegative individuals (0.6%). While the vaccine poses little serious risk, advance notification is still required [62].

### 4.2. Preclinical and Clinical Trials of CHIKV Vaccines

The inactivated vaccine BBV87 was prepared by inactivating whole-virus particles of the ECSA genotype CHIK/03/06 strain isolated in India in 2006 [50]. Phase III clinical trials showed that neutralizing antibody titers peaked 6–8 weeks after vaccination. Multiple doses ensured protective efficacy, and the vaccine also provided cross-protection against multiple CHIKV genotypes. The core advantages are high safety, suitability for immunocompromised populations, clear vaccine components, stability, and easy storage, transportation, and supervision. Nonetheless, it has some shortcomings, such as low immunogenicity, the need for multiple immunizations, and the reliance on large cell cultures for production, leading to inefficiency and high costs [49].

The live attenuated CHIKV-NoLS vaccine retains viral replication capability, albeit at an attenuated rate, and thus effectively induces a robust immune response [56]. The development of this vaccine is based on modifying the N-terminal nuclear localization sequence (NLS) in the CHIKV capsid protein. Specifically, mutations in the NLS significantly reduce the capsid protein’s nuclear entry efficiency, thereby attenuating viral replication [57]. The NLS vaccine includes the RNA genome of attenuated CHIKV, along with the CAF01 adjuvant. The RNA genome is then translated in the vaccinated individual to produce a viral antigen and initiate attenuated viral replication. The CAF01 adjuvant maintains antigenic stability and increases immune response. Preclinical animal experiments showed that a single immunization could induce persistent neutralizing antibodies and cellular immune memory [58]. Also, the CAF01 liposome delivery system can directly deliver the RNA genome in vivo, providing technical support for large-scale vaccine production [59]. The vaccine has not yet entered clinical trials, and its safety and adjuvant production need to be verified.

CHIKV subunit vaccines are developed by targeting the CHIKV E2 envelope glycoprotein, a key target for neutralizing antibodies [60]. The expression efficiency of CHIKV E2 protein in prokaryotic systems was raised via codon optimization and produced through recombinant plasmid construction, protein expression, and purification. Although protein expression has been identified and antigenic activity has been completed, immunogenicity and in vivo protective effects still need to be verified through animal and human trials.

Recombinant viral vector-based vaccines stimulate immune responses by modifying specific viral genomes to insert and express CHIKV antigen-encoding genes. The replication-deficient chimpanzee adenovirus vector vaccine utilizes a chimpanzee adenovirus, to which there is low immunity in the human population, as the vector. After knocking out the replication gene and inserting the CHIKV antigen gene, it elicited strong immunity with a single dose in mouse experiments [63]. Measles virus vectored (MV)-CHIKV vaccine uses a live attenuated measles virus as a vector to express CHIKV-like particles comprising capsid and envelope structural proteins [64]. Phase II clinical trials showed that two doses of the vaccine could induce specific antibodies with good safety [65]. The former is in the preclinical stage, while the latter requires further assessment of long-term immune persistence [66].

The EILV/CHIKV chimeric vaccine uses insect-specific Eilat virus (EILV) as the backbone and replaces its structural protein genes with the corresponding gene of CHIKV. The chimeric virus can replicate in insect cells, but because it cannot replicate in mammalian cells, it was considered non-pathogenic [69]. Animal experiments showed that it could activate T cells, memory B cells, and antibody responses in a dose-dependent manner. Moreover, it achieved cross-neutralization of multiple lineages of CHIKV [67,68], and it induced multiple innate immune pathways, including TLR signaling, antigen-presenting cell activation, and NK receptor signaling, providing rapid protection with outstanding safety [70]. Long-term safety needs to be validated in primate models, and clinical translation is required.

The nucleic acid vaccine MRNA-1388, which is the first CHIKV RNA vaccine, synthesizes mRNA encoding CHIKV antigen through in vitro transcription. Encapsulated and protected with lipid nanoparticles (LNPs), the vaccine is delivered to host cells to express antigen and trigger an immune response. Phase I clinical trials showed good safety at all doses, rapid induction of long-lasting neutralizing antibodies, immunogenicity comparable to that of live attenuated vaccines, and no significant safety issues after vaccination [71]. However, it had poor mRNA stability and required cold chain transportation, and long-term safety data are lacking.

## 5. Drug Development Strategies for CHIKV

### 5.1. Viral Entry Inhibitors

Virus entry is the key rate-limiting step in initiating infection, so inhibitors targeting this process constitute the core of an early intervention strategy. Their mechanisms of action focus on two main aspects: preventing viral particles from binding to host cell receptors and interfering with pH-dependent virus–endosomal membrane fusion (Table 2).

Attachment inhibitors: Flavagline (FL) series compounds like FL23 and FL3 effectively inhibit CHIKV attachment by targeting the host cell membrane protein Prohibitin-1 [73,74]. Studies indicate that these compounds, including sulfonamide 1M, can only block virus entry before infection and are ineffective after infection, confirming their specificity [75]. Suramin, a multivalent anionic compound, effectively inhibits the binding of the virus to the cell surface of sulfated glycosaminoglycans and directly intercalates into the virus E1/E2 heterodimer to interfere with its function [73,76]. Additionally, suramin inhibits both early virus–cell attachment and membrane fusion by targeting the CHIKV E2 protein. It should be noted that mutations in E2 protein N5R/H18Q could cause CHIKV to acquire specific resistance and affect suramin’s inhibitory effect on fusion [77]. However, suramin also shows inhibitory synergism with epigallocatechin gallate (EGCG) in vitro and can effectively block CHIKV envelope-mediated gene transfer [78,79].

Endosome fusion inhibitors: Chloroquine and hydroxychloroquine effectively inhibits the membrane fusion function of CHIKV E1 protein by increasing endosomal pH [73,74,76,78]. Studies report that chloroquine effectively inhibits CHIKV in a dose-dependent manner at concentrations of 5–20 μM, with a clear therapeutic window. It is most effective within 1–3 h post-infection, significantly reducing viral infection rates by blocking early CHIKV invasion to exert preventive effects [80]. However, the clinical efficacy of these compounds does not meet expectations, highlighting differences between in vitro models and complex human environments [76,78].

Multi-mechanism entry inhibitors: Berberine, Emodin, and their derivatives inhibit CHIKV invasion by downregulating the host receptor Mxra8 or delaying endosomal acidification [81]. Berberine inhibits CHIKV replication and exhibits broad-spectrum antiviral activity (EC_50_ = 1.8 μM), exerting significant antiviral and anti-inflammatory effects by inhibiting the mitogen-activated protein kinase (MAPK) signaling pathway (ERK, p38, JNK) activated by CHIKV infection [82,83]. EGCG blocks cell attachment by binding to viral surface proteins to inhibit pseudovirus entry, viral replication, and progeny production while exerting direct antiviral effects at multiple stages of CHIKV infection [73,76,81]. Abidol inhibits viral attachment and entry at an early stage while destroying the endosomal/lysosomal membrane structure to prevent viral release, and its drug-resistant mutation G407R was located in the E2 protein of CHIKV [73,84]. Imipramine and U18666A effectively inhibit viral entry by interfering with intracellular cholesterol transport and disrupting the lipid environment required for viral fusion [73,84].

**Table 2:** Viral entry inhibitors.

Drug Names	Mode of Action	Median Effective Concentration EC50 (μmolL^−1^)	Median Cytotoxic Concentration CC50 (μmolL^−1^)	Reference
FL23	Inhibition of adhesion (targeting Prohibitin-1)	0.21 ± 0.03	8.9 ± 0.5	[73,74,75]
FL3		0.17 ± 0.02	7.8 ± 0.4	[73,74,75]
FL26		0.28 ± 0.04	10.1 ± 0.7	[73,74,75]
FL27		0.31 ± 0.03	12.4 ± 0.8	[73,74,75]
FL28		0.25 ± 0.02	9.5 ± 0.6	[73,74,75]
FL29		0.33 ± 0.05	13.2 ± 1.1	[73,74,75]
Suramin	Inhibition of attachment and membrane fusion (targeting E2 protein)	4.3 ± 0.3	>200	[73,76,77,78,79]
Chloroquine/hydroxychloroquine	Inhibition of endosomal fusion (increase in endosomal pH)	8.8 ± 0.7	>100	[73,74,76,80]
Berberine	Downregulation of Mxra8 receptor and delay of endosomal acidification	1.8 ± 0.2	>100	[74,81,82,83]
Emodin and its derivatives		6.1 ± 0.4	>100	[74,81]
EGCG	Binds to viral surface proteins and blocks attachment	8.7 ± 0.7	>100	[73,74,76]
Abidol	Inhibition of attachment and entry; destroys the endosomal membrane	IC_50_ ≈ 5–10 µg/mL(10–20 μmol/L)	n.s.	[73,84]
Imipramine	Interferes with cholesterol transport	2.1 ± 0.3	>100	[73,84]
U18666A		1.3 ± 0.2	>100	[73,84]

Abbreviations: FL: flavagline; Mxra8: matrix remodeling-associated protein 8; EGCG: epigallocatechin gallate. n.s.: not defined

### 5.2. Viral Replication and Gene Expression Inhibitors

This strategy focuses on the core complex of CHIKV replication, including direct targeting of virus-encoded nsPs and exploitation of host-dependent factors  (Table 3).

nsP1 inhibitors: nsP1 is a key factor in CHIKV replication responsible for catalyzing the synthesis of the 5′cap structure of viral RNA. Its dual enzymatic activity as a guanylate transferase and methyltransferase makes it an important antiviral target [85]. Based on their mechanisms of action, nsP1 inhibitors can be categorized into three classes. The MADTP and CHVB series directly inhibit the activity of MTase and GTase by binding to S-adenosylmethionine (SAM) catalytic sites. The 6′-fluoro-homoneplanocin A (FHNA) series, on the other hand, interferes with the membrane binding and oligomerization of nsP1 by targeting the secondary binding pocket of the *r*ing-*a*perture *m*embrane-*b*inding and *o*ligomerization domain without directly inhibiting enzymatic activity [86]. Lead compounds, such as pyrimidine derivatives, guanosine triphosphate (GTP), and nucleoside analogues, are shown to significantly inhibit nsP1, suppressing CHIKV replication [87]. The nsP1-targeted MADTP-resistant strain still maintains mosquito-borne transmission efficiency comparable to that of the wild-type strain and stably retains the resistant genotype in saliva, suggesting that drug development for this target should address the risk of resistance [88].

nsP2 inhibitors: nsP2 plays a key role in CHIKV replication and is responsible for processing viral multiprotein precursors. A variety of compounds, such as ID1452-2, Bassetto, and Compound 1, have inhibitory activity against nsP2 [73,76,78]. The nsP2 inhibitor RA-0002034 can efficiently and selectively inhibit CHIKV replication by modifying the cysteine residue in nsP2 [89]. The natural compound Withaferin A (WFA) effectively blocks CHIKV multiprotein processing and RNA synthesis by binding to nsP2 and utilizing its oxidative properties to inhibit enzymatic activity, which could be reversed by reducing agents [90]. J12/J13, as novel CHIKV nsP2 inhibitors, effectively inhibit CHIKV replication in cell models, with J13 also exhibiting good oral bioavailability [91]. The antihypertensive drug telmisartan and neomycin can inhibit CHIKV replication at low molar concentrations by binding to nsP2 and inducing conformational changes, showing potential as anti-CHIKV agents [92]. Heat shock protein 90 (Hsp90) promotes CHIKV protein synthesis by directly stabilizing nsP2 and activating the Akt/mTOR signaling pathway. Its inhibitor, geldanamycin, and its second-generation analogue, 17-AAG, effectively inhibit viral replication by disrupting Hsp90–nsP2 interaction and blocking the signaling pathway [93].

nsP3 inhibitors: nsP3 has multiple functions throughout the CHIKV life cycle, with a structure consisting of a macro domain, an AUD, and an HVD [94]. Among pyrazinazole compounds, the lead compound, CMPD 104, exhibits high binding affinity and favorable drug-like properties for the CHIKV nsP3. This compound, which can form a stable complex with CHIKV nsP3, shows potential for the development of nsP3-targeted anti-CHIKV drugs [95]. Derivatives B1 and B7, based on Lomerizine’s structure, exhibit significant anti-CHIKV activity, and their mechanisms of action are closely related to targeting viral nsP3. B1 can stably bind to nsP3’s active site and exhibits favorable absorption, distribution, metabolism, excretion, and toxicity (ADMET). This series of compounds provides a novel strategy for the development of anti-CHIKV drugs targeting nsP3 [96]. With less or no side effects, natural compounds in purified or crude extract forms could be used as preeminent and safe therapies against CHIKV infection. Harringtonine exerts an anti-CHIKV effect by blocking early viral replication by inhibiting the translation of key viral proteins, such as nsP3 and E2. It exhibits inhibitory activity against a variety of alphaviruses, demonstrating broad-spectrum antiviral potential [76,97].

nsP4 inhibitors: nsP4, as a core RdRp for CHIKV replication, is an important target for nucleoside antiviral drugs. The conserved C-terminal RdRp domain is responsible for CHIKV genomic and subgenomic RNA synthesis, and N-terminal adenylyltransferase activity is involved in the formation of the poly(A) tail. Nucleoside drugs, such as favipiravir and sofosbuvir, inhibit CHIKV replication by mimicking natural nucleotides that, once inside of the cell, are metabolized and then integrated into the nascent RNA strand, triggering chain termination or fatal mutations [73,76,78]. Compound A, as a benzimidazole derivative, can inhibit multiple CHIKV strains at nanomolar concentrations. The methionine residue at position 2295 (M2295) in the RNA polymerase functional domain of nsP4 is a key target. Compound A exerts antiviral effects by inhibiting RdRp function, effectively suppressing multiple CHIKV strains [98]. The ribonucleoside analogue 4′-fluorouridine (4′-FlU) suppresses CHIKV replication by inhibiting the RdRp function of nsP4. Oral administration significantly reduces CHIKV viral load and alleviates symptoms, showing potential for the development of anti-CHIKV drugs [99,100]. In addition, the cobalt (III) thiosemicarbazone complex exerts a potent anti-CHIKV effect by targeting CHIKV nsP4 and acting during the post-replication stage [101].

**Table 3:** Inhibitors of viral replication and gene expression.

Drug Names	Mode of Action	Median Effective Concentration EC50 (μmolL^−1^)	Median Cytotoxic Concentration CC50 (μmolL^−1^)	Reference
MADTP-314	Inhibition of nsP1 (targeting SAM site)	0.8 ± 0.1	>100	[85,86]
MADTP-372		0.5 ± 0.1	>100	[85,86]
FHNA series	Suppression of nsP1 (interference with membrane binding)	n.s.	n.s.	[86]
Pyrimidine derivatives/GTP/nucleoside analogues	Inhibition of nsP1	n.s.	n.s.	[87]
6-azauridine		3.2 ± 0.3	>20	[87]
ID1452-2, Bassetto, Compound 1	Inhibition of nsP2	n.s.	n.s.	[73,76]
RA-0002034		0.9 ± 0.1	>100	[89]
WFA		0.6 ± 0.1	>50	[90]
J12		0.7 ± 0.1	>100	[91]
J13		0.5 ± 0.1	>100	[91]
Telmisartan		8.3 ± 0.6	>200	[92]
Novobiocin		5.7 ± 0.4	>200	[92]
Geldanamycin	Inhibition of Hsp90 (disruption of Hsp90–nsP2 interaction)	0.08 ± 0.01	2.1 ± 0.2	[93]
17-AAG		0.12 ± 0.02	4.5 ± 0.3	[93]
CMPD 104	Inhibition of nsP3	1.4 ± 0.1 >100	1.4 ± 0.1 >100	[95]
Derivatives B1 Derivatives B7		n.s. n.s.	29.72 31.68	[96]
HT	Inhibition of nsP3/E2 protein translation	0.24	2.04	[97]
Favipiravir	Inhibition of nsP4 (nucleoside analogue)	n.s.	n.s.	[73,76,78]
Sofosbuvir		n.s.	n.s.	[73,76,78]
Compound A	Inhibition of nsP4 RdRp	3.1	>50	[98]
4′-FlU	Inhibition of nsP4 RdRp	0.3–0.42	>100	[99,100]
Cobalt(III) thiosemicarbazone complex	Inhibition of nsP4	2.97	420	[101]

Abbreviations: SAM: S-adenosylmethionine; FHNA: 6′-fluoro-homoneplanocin A; nsP: nonstructural protein; GTP: guanosine triphosphate; WFA: Withaferin A; Hsp90: heat shock protein 90; HT: Harringtonine; RdRp: RNA-dependent RNA polymerase; 4′-FlU: 4′-fluoruridine.n.s.: not defined

### 5.3. Host-Targeted Antiviral Therapy (HDAT)

HDAT aims to reduce the risk of viral drug resistance by targeting the host factors necessary for viral replication with broad-spectrum antiviral potential  (Table 4).

Regulating nucleotide metabolism is a key strategy for combating CHIKV. Ribavirin works by depleting the GTP pool and mis-incorporating into RNA, while mycophenolic acid interferes with viral nucleic acid synthesis by inhibiting inosine monophosphate dehydrogenase (IMPDH) [73,76,78]. In vitro studies show that ribavirin in combination with IFN-α exerts a potent synergistic effect, reducing CHIKV viral load at clinical concentrations [102]. This combination also shows synergistic effects in DENV studies, inhibiting most DENV replication [103]. Mycophenolic acid (MPA) can inhibit CHIKV replication and block CHIKV-induced apoptosis by suppressing IMPDH to deplete the intracellular GTP pool [104].

Inhibiting Hsp90’s function is an effective strategy against CHIKV. Geldanamycin and its derivatives (HS-10, SNX-2112) disrupt the stability of viral replication complexes by inhibiting Hsp90 and inducing the degradation of viral nsPs, such as nsP2 and nsP3. Hsp90 supports the assembly and function of viral replication complexes by maintaining the proper folding and stability of viral proteins through its molecular chaperone function [73,76,78]. The specific interactions between Hsp90 and nsP3 and nsP4 are crucial for viral replication. Inhibiting Hsp90 function can effectively block CHIKV replication, exerting an anti-CHIKV effect [105]. A mechanistic study showed that Hsp90 interacted with CHIKV nsP2 and activated the PI3K/Akt/mTOR signaling pathway to promote CHIKV mRNA translation, playing a key role in early CHIKV replication. Different CHIKV strains have varying sensitivities to Hsp90 inhibitors, such as geldanamycin, which offers new insights for the development of anti-CHIKV drugs. Silvestrol inhibits CHIKV replication by specifically inhibiting the eukaryotic initiation factor eIF4A to block the translation of CHIKV mRNA [76,78]. Another study shows that the sphingosine kinase inhibitor SLL3071511 exhibits anti-CHIKV activity in vitro, further suggesting that this target has host-directed anti-CHIKV therapeutic potential [106].

### 5.4. Innate Immune Response Activators

Strengthening the host’s innate antiviral defenses faces genetic barriers, with a lower likelihood of inducing drug resistance.

RIG-I/MDA5 Pathway: In the early stage of CHIKV infection, RIG-I within host cells acts as a core innate immune recognition receptor, recognizing the 3′-UTR of the CHIKV genome and initiating an early host antiviral response [107]. Melanoma differentiation-associated gene 5 (MDA5) does not exhibit significant binding to the viral genome [108]. However, during acute CHIKV infection, the host’s innate immune system is significantly activated. This results in the activation of MDA5, along with subsequent IL-12 and IFN-α responses, in association with more effective CHIKV clearance, reflected by reduced CHIKV viral load [109]. CHIKV inhibits MDA5/RIG-I-mediated NF-κB activation through nsP2, E1, and E2, interfering with type I IFN production and significantly suppressing the MDA5 pathway [110]. This cascade suppresses innate immune defenses, allowing the virus to escape surveillance.

cGAS-STING Pathway Agonists: The synthetic molecules dimeric amidobenzimidazole (diABZI) and cyclic adenosine monophosphate–inosine monophosphate (cAIMP) exhibit preventive and therapeutic potential by activating the STING-dependent type I IFN pathway [107]. In CHIKV infection, the host senses cytoplasmic DNA following infection through cGAS and activates the STING pathway to limit CHIKV replication [111]. Systematic screening shows that STING agonists, including cAIMP, diABZI, and 2′,3′-cGAMP, and the Dectin-1 agonist hard-core glycan all have broad-spectrum antiviral properties and can inhibit a variety of RNA viruses, including CHIKV. cAIMP treatment reverses CHIKV-induced dysregulation of cell repair, immune, and metabolic pathways and provides protection in mouse models of chronic CHIKV-induced arthritis [112]. Additionally, cAIMP activates the STING-TBK1-IRF3 axis, induces a robust type I IFN response, and reshapes cellular metabolism, effectively inhibiting CHIKV replication in both cellular and mouse models. Its effect is superior to that of the direct-acting antiviral drug remdesivir, establishing the potential of STING agonists as host-directed anti-CHIKV strategies [107].

TLR agonists: TLR agonists act as innate immune activators and play a significant role in CHIKV infection. During CHIKV infection, TLR3 mainly plays a protective antiviral role, while TLR4 is exploited by the virus to facilitate its entry into the host cell. The activation of TLR triggers a robust cytokine storm, which is associated with acute and chronic symptoms of the disease, such as arthralgia [113]. Polyinosinic acid: polycytidylic acid [poly(I:C)] is a synthetic analog of double-stranded RNA that acts as a potent TLR3 agonist. Pre-clinical studies have consistently shown that poly(I:C) is well-tolerated at the doses employed for vaccination. Poly(I:C) and Imiquimod (TLR7 ligand) activate the NF-κB and IRF pathways by viral mimicry of nucleic acids, inducing type I IFN production [107]. Studies have found that CHIKV inhibits the innate immune response mediated by the TLR3/TLR7 pathways, resulting in changes in the expression levels of TLRs, antiviral genes, and cytokines. In contrast, poly (I:C) inhibits CHIKV replication by upregulating the TLR3 pathway and related antiviral genes, highlighting its potential as an anti-CHIKV drug [114,115]. In addition, poly (I:C) shows significant inhibitory effects on CHIKV replication in human bronchial epithelial cells [116].

### 5.5. Novel Therapeutic Targets and Strategies

With in-depth investigation of CHIKV pathogenesis, new therapeutic targets continue to be discovered, offering important directions for the development of anti-CHIKV drugs.

Natural products have been an important source of anti-CHIKV drugs. Among them, salidroside exerts anti-inflammatory and cell-protective effect through the synergistic effects of multiple pathways at the cellular level by regulating multiple targets, such as TNF, IL-6, NFKB1, mediating the PI3K-Akt, NF-κB and MAPK signaling pathways, and inhibiting the activity of GPX4 [117]. In addition, flavonoids, such as Baicalein and Fisetin, alkaloids like Harringtonine (EC_50_ = 0.24 µM), and diterpenoids like Trigocherrierins can effectively inhibit the key links of viral replication, showing good anti-CHIKV activity [118,119]. Curcumin, a natural product, can relieve pain and improve symptoms of acute and chronic arthritis by maintaining cartilage integrity and reducing the loss of proteoglycans and type II collagen [120].

Regulation of the microbiota–metabolic axis: The microbiota–metabolic axis regulates CHIKV infection and joint inflammation. In addition to typical symptoms, CHIKV infection can be accompanied by gastrointestinal manifestations. The gut microbiota has an anti-CHIKV protective effect with depleted microbiota, increasing viral load, inflammation, and tissue damage. Microbiota deficiency weakens the TLR7-MyD88 signaling pathway in pDC cells and inhibits type I IFN production [121].

**Table 4:** Host targeting, immunomodulation, and other antiviral drugs.

Drug Names	Mode of Action	Median Effective Concentration EC50 (μmolL^−1^)	Median Cytotoxic Concentration CC50 (μmolL^−1^)	References
Ribavirin	Exhaustion of GTP, mis- incorporation into RNA	100.5	786.6	[73,76,78,102]
MPA	Inhibition of IMPDH and depletion of GTP	0.56	65.2	[73,76,78,104]
HS-10	Suppression of Hsp90	0.15 ± 0.02	6.2 ± 0.5	[73,76,78,93,105]
SNX-2112		0.09 ± 0.01	3.8 ± 0.3	[73,76,78,93,105]
Silvestrol	Inhibition of eIF4A	n.s.	n.s.	[76,78]
Sphingosine kinase inhibitor (SLL3071511)	Inhibition of sphingosine kinase	2.1	16.2	[106]
diABZI	cGAS-STING pathway agonists	0.16	>10	[107]
cAIMP		1.2	>100	[107,112]
2′,3′-cGAMP		3.5	>100	[112]
Hard-core glycans	Dectin-1 agonist	~0.011 × 10^−3^	>0.083 × 10^−3^	[112]
Poly(I:C)	TLR3 agonist	n.s.	n.s.	[107,114,115,116]
Imiquimod	TLR7 agonist	n.s.	n.s.	[107]
Salidroside	Multi-target, anti-inflammatory, cell-protective	25.3	>200	[117]
Flavonoids (e.g., Baicalein, Fisetin)	Inhibition of viral replication	n.s.	n.s.	[118,119]
Diterpenoids (Trigocherrierins)		3.7 (Trigocherrierin A)	89.2 (Trigocherrierin A)	[119]
Harringtonine		0.24	2.04	[119]
MTX	Immunomodulatory therapy	n.s.	n.s.	[122,123,124,125]
Etanercept	anti-TNF therapy	n.s.	n.s.	[126,127,128]

Abbreviations: GTP, guanosine triphosphate; Hsp90, heat shock protein 90; eIF4A, eukaryotic initiation factor 4A; MPA, mycophenolic acid; IMPDH, inosine monophosphate dehydrogenase; diABZI, dimeric amidobenzimidazole; cGAS-STING, cyclic GMP-AMP synthase-stimulator of interferon genes; cAIMP, cyclic adenosine monophosphate-inosine monophosphate; Poly(I:C), polyinosinic acid: polycytidylic acid; TLR, Toll-like receptor; MTX, Methotrexate. n.s.: not defined

Immunomodulatory therapy: Methotrexate (MTX), an immunomodulatory agent, is a commonly used disease-modifying antirheumatic drug for chronic arthritis following CHIKV infection [122]. It demonstrates significant clinical efficacy, with studies showing that MTX monotherapy or combination therapy improved symptoms in patients, achieving partial recovery in 75% of cases and showing better response in seropositive patients [123]. Although its mechanisms are not fully understood, current evidence suggests that its action may be independent of the Prostaglandin E2 (PGE2)-mediated inflammatory pathway. In clinical practice, MTX is widely used to control CHIKV-induced immune arthritis, showing synergistic efficacy when combined with hydroxychloroquine [124]. It is currently listed as a core treatment option for CHIKV-related arthritis in multiple guidelines, with consensus on its safety, cost-effectiveness, and accessibility, though more randomized controlled trials are needed to further verify its long-term efficacy [122]. Anti-TNF biologics are monoclonal antibodies or receptor fusion proteins that treat autoimmune diseases by specifically neutralizing tumor necrosis factor-alpha (TNF-α) to suppress excessive immune responses. Research indicates that while anti-TNF therapy does not increase the risk of CHIKV infection, it significantly reduces symptom chronicity and alleviates joint damage [126]. Etanercept can relieve joint pain and inflammation but may also delay viral clearance, suggesting that clinical application requires weighing anti-inflammatory benefits against potential risks and considering use after the acute phase or in combination with antiviral drugs [127]. However, in clinical practice, for patients with stable rheumatoid arthritis (RA), CHIKV infection can render their existing anti-TNF therapy (such as etanercept) ineffective, often necessitating an escalation of immunosuppressive regimens. This includes increasing glucocorticoid doses or switching to/adding other second-line biologics (such as adalimumab and rituximab) [128]. This indicates that CHIKV infection can breach existing immune control, frequently leading to an upgrade in treatment strategy.

## 6. Conclusions and Future Perspectives

As Aedes mosquitoes’ range expands and climate change progresses, the global prevalence of CHIKV poses a threat to public health. Although progress has been made in detecting CHIKV, these technologies still face multiple challenges that hinder their rapid and accurate global application. As the gold standard test for CHIKV, RT-PCR imposes strict requirements in terms of the laboratory environment, equipment, and operators, and it is time-consuming and relatively expensive. These limitations make it difficult to utilize in CHIKV-endemic developing countries and remote areas. These limitations also hinder rapid, large-scale screening during outbreaks [129]. At present, point-of-care testing (POCT) technologies, such as rapid diagnostic kits, are convenient and fast, but their sensitivity and specificity are sometimes lower than those of laboratory methods, especially when the viral load is low or in the later stages of infection, when false negative or false positive results may occur [130]. CHIKV shares high homology in antigenic epitope regions with other members of the same genus, such as DENV and ZIKV [131]. Because the monoclonal or polyclonal antibodies employed in POCT reagents are prone to cross-reactivity with the aforementioned homologous viruses, false-positive results are induced. Currently available CHIKV POCT technologies are predominantly based on qualitative detection, only providing semi-quantitative outcomes, such as “positive,” “negative,” or “weakly positive”, and failing to achieve accurate quantitative analysis of viral load or antibody titer in samples. Notably, viral load is closely correlated with the severity of clinical symptoms caused by CHIKV infection. Furthermore, detection signals are susceptible to interference from substances in samples, such as proteins and metabolites, which can prolong detection [132]. Additionally, CHIKV may continue to mutate, so the effectiveness of existing detection reagents, particularly primers, probes, and antibodies, in detecting emerging viral variants needs to be monitored and assessed. Looking ahead, it is expected that POCT technology will gradually gain wider adoption. These devices combine sample processing, nucleic acid amplification, and result detection, enabling highly sensitive and specific viral nucleic acid testing to be carried out in a short time, even in resource-challenged environments. On the other hand, rapid test kits that require no nucleic acid extraction and provide visual readouts are suitable for primary care institutions and home use, where specialized equipment is unavailable [133]. In addition, with AI technology, future testing devices may be connected to smartphone apps to enable automatic interpretation of test results and real-time epidemic reporting, building a powerful regional data network to provide data support for global epidemic prevention and control.

CHIKV prevention and control also face challenges. Seroepidemiologic and animal model evidence, as well as neutralizing antibody titers, are the core protective test indicators for CHIKV vaccines. Vaccines based on one strain may exhibit cross-protection against other genotypes, but studies have shown differences in infection and immune mechanisms between different lineages, as well as significant differences in serum neutralizing antibody levels [134]. Thus, CHIKV vaccines struggle with the genetic diversity of the virus and reliable immune correlates of protection. Future research and development should focus on three directions. First, based on E1/E2 protein structure data, antigens can be optimized through multi-epitope fusion and conformational modification in combination with AI to predict viral variations to develop broad-spectrum vaccines that cover mainstream genotypes [135]. For mRNA vaccines, a novel LNP vector validated by COVID-19 vaccines can be used to strengthen the vaccine’s stability and reduce cold chain dependence [136]. Second, research and development need to be optimized for different populations. For example, low-toxicity adjuvant formulations should be developed for infants and young children, safety grading assessment standards should be established for pregnant women, and enhanced immunity should be engineered for older people, along with optimizing production and costs through technological innovation to improve accessibility [137]. Third, vaccine production capacity can be expected to increase through cell-free synthesis, continuous flow culture, and other technologies [138]. At the same time, neurotoxicity biomarkers will be screened based on IXCHIQ adverse event data, a long-term safety monitoring network will be constructed, and, ultimately, a scientific and efficient CHIKV prevention system will be formed.

As of the end of 2025, no specific antiviral drugs for CHIKV have been approved, and clinical management remains supportive, including fever control, analgesia, and fluid replacement. Worldwide research is focused on two broad tracks: direct-acting virus-targeted inhibitors and host-factor-targeted interventions. Overall, CHIKV antiviral development is still in the “proof-of-concept to early in-vivo” phase. Drug repurposing offers the most accessible near-term option, whereas structure-guided novel molecules and combination therapies directed at both viral and host targets represent the mid- to long-term path forward.

## Figures and Tables

**Table 1:** Vaccines for the prevention of CHIKV infection and their research and development progress.

Vaccine Type	Vaccine Name	Antigens	Research and Development Stage	Results	Advantages	Disadvantages	References
Inactivated vaccine	BBV87	C, E1, E2, E3, 6K, nsP1, nsP2, nsP3, nsP4 proteins	Phase III clinical	Neutralizing antibodies peaked 6 to 8 weeks after vaccination and were generally well tolerated	High safety, suitable for immunocompromised populations; stable and easy to store, with clear and regulated ingredients	Low immunogenicity requires multiple immunizations; production requires a large number of cell cultures, which is inefficient and costly	[49,50]
Live attenuated vaccine	IXCHIQ (VLA1553)	C, E1, E2, E3, 6K, nsP1, nsP2, nsP3, nsP4 proteins	No longer approved in the U.S.	A phase III clinical trial (4115 healthy participants) showed a serum positive conversion rate of 98.9%, maintaining protective threshold antibody levels after 180 days and allowing for a serious adverse event rate	The world’s first CHIKV vaccine to be approved; single-dose immunization; strong immunogenicity, inducing long-lasting immunity; it can cross-protect against multiple related viruses	There is a risk of restoring pathogenicity, and it is not suitable for people with weakened immunity	[48,51,52,53,54,55]
	CHIKV-NoLS (CAF01 delivery)	C, E1, E2, E3, 6K, nsP1, nsP2, nsP3, nsP4 proteins	Preclinical	Animal experiments showed long-acting single-dose immunization preparation effects	A single dose can provide long-term protection and overcome problems of insufficient antibody levels and limited immune memory associated with traditional single-dose strategies	Safety needs to be further verified; large-scale application of the delivery system (CAF01) remains to be evaluated	[56,57,58,59]
Subunit vaccine	pET-28aCHIKV E2 recombinant protein vaccine	E2 protein	Preclinical	Recombinant plasmids were successfully constructed, and fusion proteins were expressed	Codon-optimized to enhance protein expression efficiency; provides candidate materials for the development of E2 protein-related subunit vaccines	Only protein expression and identification are completed; further verification of immunogenicity and protective effect is required	[60]
VLP vaccine	VIMKUNYA (PXVX0317)	C, E1, E2 proteins	Approved for marketing (U.S., E.U., etc.)	Good safety at all doses in clinical studies; no serious adverse events; the neutralizing antibody response was rapid and durable after vaccination	No risk of infection, high safety; mimics the conformation of a natural virus; good immunogenicity; it works without adjuvants and is easy to inoculate	The protective effect for special groups, such as children, remains to be verified; production processes need to be optimized to enhance global supply capacity	[61,62]
Recombinant viral vector-based vaccine	Copy-defective chimpanzee adenovirus vector vaccine	C, E1, E2, E3, 6K proteins	Preclinical	Animal experiments showed significant single-dose immune effects	The pre-immunization rate is low; a single dose can trigger a strong immune response	Further research is needed to confirm clinical efficacy; the mass production process of the carrier needs to be optimized	[63]
	MV-CHIK (measles virus vector)	E1, E2, E3 proteins	Phase II clinical	Specific neutralizing antibodies were detected after two intramuscular injections, and no serious adverse events occurred	Good clinical performance; high safety	Long-term immune persistence needs to be evaluated; studies for special populations, such as children, are still ongoing	[64,65,66]
Chimeric vaccine	EILV/CHIKV (Eilat virus chimerism)	E1, E2, E3 proteins	Preclinical	Animal experiments showed cross-neutralizing effects on CHIKV lineages from Asia, West Africa, and the Indian Ocean; Guinea pigs showed no skin hypersensitivity reactions	High safety; no risk of regaining pathogenicity; cross-neutralizing multilineage CHIKV; activates innate + adaptive immunity and protects quickly	Relying on insect-specific viral vectors requires validation of long-term safety in primate models	[67,68,69,70]
mRNA vaccine	mRNA-1388 (Moderna)	E1, E2, E3 proteins	Phase I clinical	Evidence of good safety and tolerability at all dose levels and long-lasting neutralizing antibody responses	Short design production cycle for quick response to the epidemic; strong immunogenicity and long-lasting neutralizing antibodies	mRNA is prone to degradation and requires a special delivery system for stability; lack of long-term safety data	[71]

## Data Availability

Not applicable.
